# Developing a Knowledge Test for a Neonatal Ethics Teaching Program

**DOI:** 10.7759/cureus.1971

**Published:** 2017-12-20

**Authors:** Gregory P Moore, Emanuela Ferretti, Thierry Daboval

**Affiliations:** 1 Children's Hospital of Eastern Ontario, The University of Ottawa; 2 Paediatrics, Children's Hospital of Eastern Ontario, The University of Ottawa

**Keywords:** medical ethics, assessment tool, knowldedge test, multiple choice questions, neonatology, perinatal

## Abstract

Objective

The innovative Neonatal-Perinatal Medicine (NPM) Ethics Teaching Program at the University of Ottawa provides NPM trainees with vital foundational knowledge required to manage ethically contentious clinical scenarios frequently encountered in practice. In this study, our aim was to develop a knowledge test to assess the impact of the NPM Ethics Teaching Program on trainees’ knowledge about ethics.

Study design

Using an iterative four-step process, we developed a test for assessing pre- and post-training knowledge of NPM ethics. We first created a blueprint of the test, identifying its purpose, length, and format. We then weighted the learning outcomes of the NPM Ethics Teaching Program sessions to determine the number of questions that would be asked to assess to each learning outcome. Next, we populated the question bank and constructed a draft test. We obtained feedback from content experts on the draft test and piloted the draft test with former trainees from the NPM Ethics Teaching Program.

Results

We developed a pre- and post-knowledge test in NPM ethics consisting of 44 multiple choice questions (MCQs), each with five response options. The test takes approximately 60 minutes to complete. It took roughly 15 months to design and pilot the NPM ethics test.

Conclusions

This test can aid in the assessment of the amount of NPM ethics gained by trainees and contribute to the identification of areas for improvement in teaching and in the overall ethics program. Further iterations of the test will allow for additional assessment of its validity and the efficacy of the teaching program. Given the lack of structured evaluative ethics teaching programs in NPM nationally, this project will act as another step towards the introduction of our NPM Ethics Teaching Program to other Canadian NPM residencies.

## Introduction

With medical and technological advances and increased survival rates among babies with extreme prematurity and complex medical conditions, difficult ethical situations frequently arise in clinical practice. To manage these situations, postgraduate trainees in Neonatal-Perinatal Medicine (NPM) must have knowledge of intricate communication skills, and ethical principles and frameworks. In recent years, the NPM Ethics Teaching Program at the University of Ottawa was redesigned [1] to better address several of the Royal College of Physicians and Surgeons of Canada (RCPSC) CanMEDS roles and work continues to ensure the program fits with the CanMEDS competency-based framework [2].

The CanMEDS framework stresses the importance of knowledge of each of CanMEDS seven roles and asserts that ethical competence requires assessment using multiple modalities including written knowledge tests [3]. Clinical competence integrates multiple resources including declarative, procedural and conditional knowledge [4]. Although competency is complex and multidimensional and may be best assessed by direct observation in clinical practice, knowledge is still the root of any competency and should be appropriately assessed [5]. Reliable assessment of trainees’ knowledge helps ensure high-quality educational programming and clinical practice [6,7]. We therefore require assessment tools to evaluate our Neonatal Ethics Teaching Program. However, tools do not presently exist for assessing NPM postgraduate trainees’ knowledge in the area of neonatal ethics [8]. While a review of assessment methods for professionalism revealed 49 tools addressing a variety of ethical issues [9], all were targeted to medical students or adult specialty trainees and were not applicable to post-graduate level of training in NPM. To date, only two published tools assessing knowledge related to NPM ethics exist to examine pediatrics residents [10,11]. Despite some questions from these two tools focusing on NPM ethics, they were developed for pediatrics residents who have notable differences in the content of their training program.

This gap in the literature and the importance of assessment to demonstrate knowledge gains highlights the need for a new tool to assess trainees’ knowledge in NPM ethics. This paper details the development of a knowledge test to measure pre- and post-training postgraduate trainees’ knowledge of medical ethics related to NPM.

## Materials and methods

The Children’s Hospital Eastern Ontario Research Ethics Board and the Ottawa Hospital Research Ethics Board approved the study.

We used a multi-step, iterative process to develop the knowledge test [12,13]. To ensure that the items on the knowledge test were of equal difficulty, we used the same items on both occasions; pre- and post-training. There is a two-year period between the administration of each of the two tests which should diminish, if not eliminate, the risk of recall of test items. Recognizing that validation is an ongoing process [14], we also employed a detailed methodology to develop several lines of validity evidence.

The study team consisted of two health education researchers with expertise in assessment of tool design, two academic neonatologists with an interest in ethics (GM, EF), a neonatologist with a Master’s degree in communicative ethics (TD), and a lawyer with a Master’s degree in bioethics (Mr. Paul Muirhead).

Step 1: Creation of the blueprint for the knowledge test

The aim of this step was to develop a blueprint or systematic outline to identify content for the pre- and post-knowledge test. To develop this blueprint, the study team planned three half-day sessions over the course of two months to discuss the purpose, length, format, and content weighting for the test. The paragraphs below provide additional information on each of these topics.

a) Identification of the purpose of the test

As the initial goal in the development process, it was necessary to clearly identify the purpose of the test and to ensure that all team members agreed upon and understood this purpose.

b) Determination of the length of the test

We then aimed to determine how many questions our test should include. Determining the appropriate length of the test necessitated that we take into consideration the purpose of the test, the amount of time allotted for it, and the implications of using multiple choice questions (MCQ) (noting the general rule that 50, well-constructed, four-option MCQs take approximately 60 minutes to complete [15]).

c) Selection of the appropriate format for the test

After considering various knowledge test formats including true-false or short answer questions, the study team confirmed that MCQs would provide adequate evidence of trainees’ knowledge learning. Although short answer questions have the advantage of making students generate answers without prompting from an MCQ’s stem or response options, MCQs were chosen because they allow incorporation of specific key learning points from the neonatal ethics teaching curriculum into the response options, they are easier to mark on repetitive iterations and they remove the negative potential for a rater’s subjective interpretation to influence the marking.

d) Weighting of the sessions’ learning outcomes and content

In order to place appropriate weight on each question asked in the knowledge test, the team considered the emphasis and teaching time that the NPM Ethics Teaching Program devoted to each session as well as each session’s learning outcomes. Three of the team members (GM, EF and TD) independently ranked the learning outcomes and then, with the assistance of the educational experts, reached consensus on the ranking through discussion and examination of dedicated teaching time to each learning outcome. In addition to the relative teaching time devoted to the learning objectives during each session, another main criterion that guided the ranking process was the perceived importance of each learning outcome for the learner to be able to competently navigate ethically challenging clinical situations. This process ensured that the test proportionately represented each learning outcome and its relevant materials [15] while also considering the clinical importance of each learning outcome for a future neonatologist.

e) Development of the blueprint for the test

To conclude Step 1, we listed the learning outcomes in their weighted order and estimated the number of questions to be assigned to each of them. This information provided the necessary details to finalize the blueprint.

Step 2: Populate the question bank for the knowledge test

Prior to developing the test items, we reviewed the literature on MCQ development to ensure that we developed high-quality MCQs and avoided question and response option flaws [16]. Recognizing that the four program instructors (GM, EF, TD, PM) had the best understanding of the learning outcomes and content that made up the sessions for the program, we asked each of them to use the blueprint, with the weighted outcomes, to brainstorm an initial set of MCQs and corresponding response options related to their respective sessions. The instructors also examined other published assessment instruments, including those from Lynch, et al.’s review [9], to see how others have assessed specific ethical concepts and, as appropriate, adapted existing items for their own purposes.

Once the instructors developed their draft questions, a Research Assistant (RA) arranged two half-day brainstorming and review sessions. The RA collated and distributed the draft questions for review prior to the first session. The objectives of these sessions were to: (a) review the draft questions and rework them as needed, (b) generate additional questions if required, and (c) construct a draft knowledge test. Once the objectives were met, the RA formatted the constructed draft test and circulated it to the research team for further review and approval.

Step 3: Obtain feedback on questions from external content experts

We invited a total of three external content experts in the domain of medical education expert, theoretical bioethics, and clinical neonatology and bioethics to review and provide feedback on the draft test. The RA emailed these three external experts written information on the program, written instructions on how to complete the review, an electronic copy of the test, and a debriefing questionnaire. We asked them to provide feedback on the clarity, understandability, and level of difficulty of the test using the debriefing questionnaire. We asked these experts to: (a) comment on the length and the time allocated for the test; (b) provide feedback on the appropriateness and relevance of the test questions and answer options, and; (c) identify any questions they felt were missing or should be removed from the test [17,18]. Finally, we invited the experts to comment directly on the knowledge test to indicate any concerns or recommend changes directly related to specific questions or answer options.

Step 4: Pilot the knowledge test with former trainees from the NPM Ethics Teaching Program

Once step 3 was concluded, we piloted the knowledge test with a convenience sample of four former trainees from the NPM Ethics Teaching Program to improve the quality and utility of the test [12]. Three program instructors (GM, EF, TD) organized a mock testing session where the pilot participants completed the knowledge test as they would if they were formally taking the test. Before the test, each volunteer participant was handed a test package that included an information letter, a consent form, written instructions on how to complete the test, the draft knowledge test, and a debriefing questionnaire. Once the participants were ready to begin the test, they were given exactly 60 minutes to complete it. After completing the test, they were asked to complete the debriefing questionnaire to provide feedback. Similar to the expert review, we asked pilot participants to provide feedback on the clarity, understandability, and level of difficulty of the test. We asked them to comment on the length of the test as well as the amount of time allocated to complete it. We also asked the pilot participants to reflect on the NPM Ethics Teaching Program and to provide feedback on the appropriateness and relevance of the test questions and answer options. Finally, we asked them to identify any questions or response options they felt were missing or should be removed from the test.

The RA entered individual test responses for each pilot participant into SPSS statistics for Windows, Version 21 and corrected them using the test answer key. The RA then developed an aggregated report indicating pilot participant responses and correct answers for each question as well as the overall test scores and completion times. The study team reviewed all test responses and flagged test items that were answered incorrectly by three or more pilot participants for further review. We used the pilot participant feedback and test results to revise and improve the knowledge test.

## Results

Step 1: Creation of the blueprint

**Figure 1:**
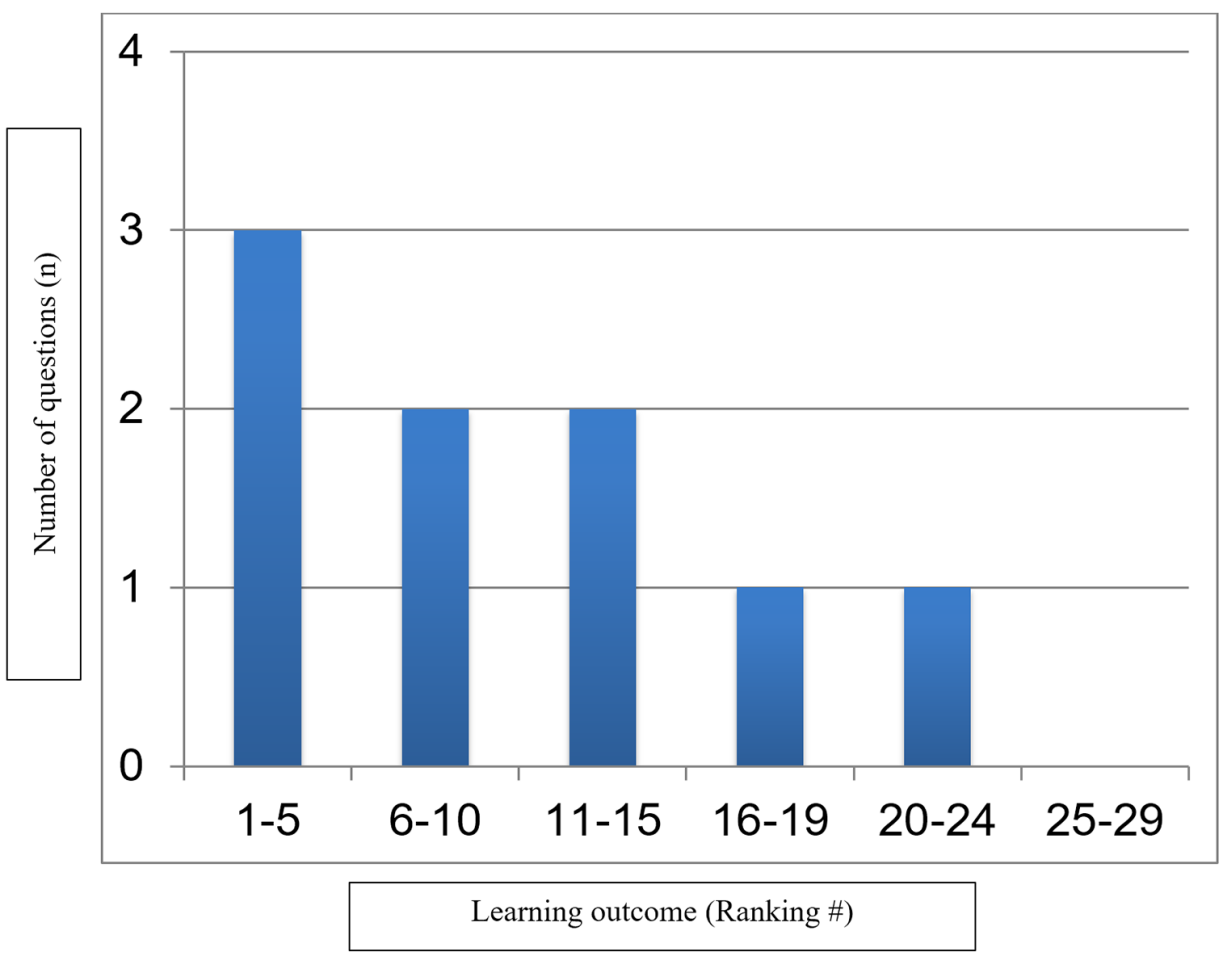
Weighted distribution of learning outcomes.

The creation of the blueprint for the knowledge test took three half-day sessions over the course of two months. Through focused discussion, we decided that the purpose of the test is to determine the amount of knowledge that postgraduate trainees gain from the NPM Ethics Teaching Program. We chose MCQ format for its suitability to the learning outcomes and its ease and consistency in scoring results; we also agreed on the 60 minutes available time for test completion. Table 1 lists the top five ranked learning outcomes of the NPM Ethics Teaching Program; Figure 1 shows the weighted distribution of the independently ranked learning outcomes.

**Table 1:** Top 5 ranked learning outcomes of NPM Ethics Teaching Program. NPM: Neonatal-Perinatal Medicine.

#	Description
1	To apply ethical approaches to clinical situations
2	To define five key bioethical principles
3	To recognize the different clinical and ethical approaches to generating care plans in neonatology
4	To describe ways to resolve disagreements regarding treatment options
5	To identify principles of good communication with patients

Step 2: Populate the question bank

It took approximately six months for the four program instructors to become familiar with MCQ development in order to create their MCQ’s and corresponding response options. At the conclusion of the two half-day sessions to review the questions and responses, the participants generated a primary version of the knowledge test that included 44 MCQs related to 24 of the 29 learning outcomes.

Step 3: External content experts review

Including the invitation process, four months were necessary for the medical education expert to provide feedback on the structure, readability and clarity of the MCQs. Table 2 summarizes the feedback from responses of the two content experts. Their written answers (not shown) to additional open-ended questions provided reasoning for the responses in the feedback form, allowing us to further improve the quality of the knowledge test.

**Table 2:** Summary of content experts’ feedback. ^*^One expert did not choose ‘agree’ or ‘disagree’ but wrote many comments suggesting they were not in complete agreement or complete disagreement. ^**^One expert did not choose ‘agree’ or ‘disagree’ but wrote ‘Variable’.

Please consider the following statements and add a checkmark under either ‘Agree’ or ‘Disagree’.		Agree	Disagree
	The test items were at an appropriate level of difficulty.	0	1*
	I found this test fair.	0	1*
	The clinical scenarios reflected my experiences in Neonatal-Perinatal Medicine.	2	0
	The test items were clear.	0	2
	The test items were easily understandable.	0	1**
	I thought the test was an acceptable length.	2	0
	The time for completing the test was adequate.	2	0

Step 4: Pilot phase

**Figure 2:**
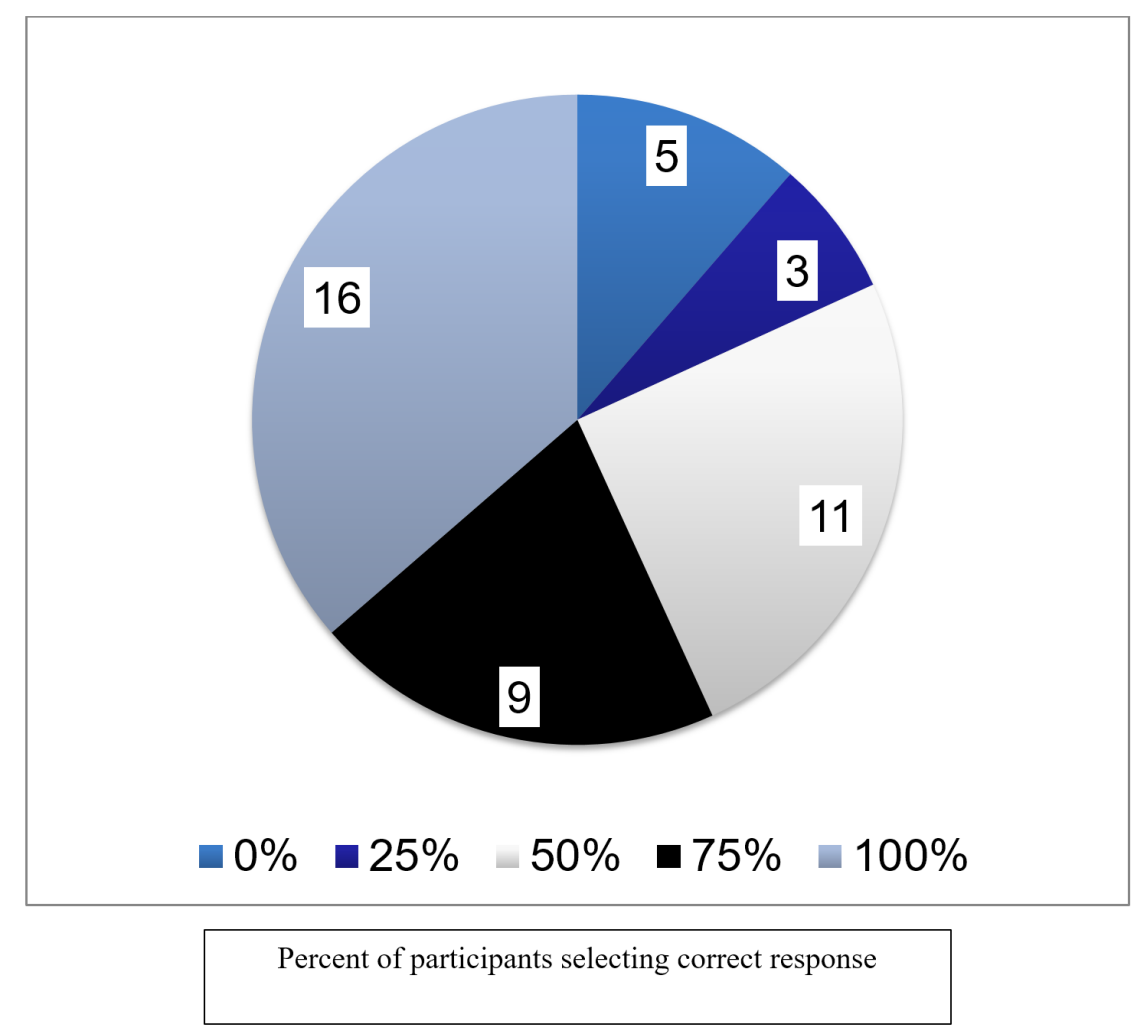
Frequency (out of 44 items) of different percentages of correct response selection by former NPM Ethics Teaching Program participants (n = 4). NPM: Neonatal-Perinatal Medicine.

Overall, the scoring percentages of the four pilot participants on the draft test were 80%, 68%, 64% and 52%, respectively. Figure 2 demonstrates the response selection of the participants. Sixteen questions were answered correctly by all four participants; five questions were not answered correctly by any of the four participants. Table 3 summarizes feedback on the test from the pilot participants. Based on the pilot participants’ response selection and their feedback, a final review of the test was completed. The review focused on the construct of the questions, grammatical errors and item-specific comments. From the review, seven of the 44 MCQs had their stem altered and 13 had one or more of their responses modified. It took two months to complete the pilot testing, to review the feedback and to create the final version of the knowledge test.

**Table 3:** Summary of pilot participants’ feedback. ^*^One participant did not choose ‘agree’ or ‘disagree’ but wrote “Some were clear” and “Some were easily understandable”.

Please consider the following statements and add a checkmark under either ‘Agree’ or ‘Disagree’.		Agree	Disagree
	The test items were at an appropriate level of difficulty.	4	0
	I found this test fair.	4	0
	The clinical scenarios reflected my experiences in Neonatal-Perinatal Medicine.	4	0
	The test items were clear.	3*	0
	The test items were easily understandable.	3*	0
	I thought the test was an acceptable length.	4	0
	The time for completing the test was adequate.	4	0

## Discussion

We believe that this systematically developed knowledge test in neonatal ethics should help to fill in, at least partially, an important gap in the literature – the lack of well-designed assessment tools to evaluate knowledge and competencies to navigate ethically sensitive situations in neonatology [3,8,19]. The test will be applicable to NPM and other training programs that share the common and relevant learning outcomes of our NPM Ethics Teaching Program and assist in assessing medical ethics knowledge in the era of competency-based medical education. The rigorous well-structured design process and the improvements made after external expert’s feedback supports the content validity of the neonatal ethics knowledge test. However, the small sample size of this pilot study did not allow for testing the reliability and construct validity of our test.

Medical educators must assess their trainees’ learning upon the completion of training initiatives, as they are accountable to these trainees and the patients and families whose care will be entrusted to them [15]. Although not the best strategy to assess a multifaceted competency, a knowledge test is necessary to ensure high-quality educational programming and clinical practice. It allows the opportunity to evaluate if the program’s curriculum is well taught [6,7] and that learners are retaining the information in, at least, the short term while they are actively participating in our program. In addition, assessment directs and influences trainees’ learning, even increasing the potential for learners to truly search for meaning and understanding [7].

The role of written tests remains important because the ability of trainees to demonstrate the understanding of key concepts is essential for their clinical competency [3,20]. Structured ethics training programs require an evaluative component including assessment tools such as written tests. Existing tools do not assess the specific knowledge in NPM ethics or meet our specific objective to evaluate the efficacy of the NPM ethics teaching program. The Socio-moral Reflection Measure of Gibbs, used by Self, et al. for first-year medical students, focuses on assessing trainees’ social-moral judgement skills [21] rather than their knowledge of key ethical concepts. Although trainees in NPM may demonstrate these judgement skills, they also require the necessary knowledge of key ethical concepts to deal with ethically challenging situations [22]. Kesselheim, et al. developed an ethics knowledge test for pediatrics residents but only five questions were related to neonatology, which was insufficient to meet our goals [8]. Many other ethics assessment tools focus on attitudes and behaviour rather than knowledge [9].

A review of 20 years of literature [9] found only nine examples of written knowledge tests. Although some of the content in these knowledge tests may be applicable to NPM trainees, they were intended for medical students (n = 7) or other adult specialty trainees (n = 2). Notably, Sulmasy, et al. [23] did create a 15-item MCQ test with face validity and a Cronbach’s alpha of 0.76-0.86, but nearly all the MCQ dealt with specific adult and/or American medico-legal issues. Another written test only assessed medical students’ reasoning in justifying an ethical decision as a means to explore knowledge gain [24].

A major strength of our study is that the test reflects our NPM ethics teaching program learning objectives and accurately measures the efficacy of our program in supporting ethics knowledge gain. There were several limitations with our test and its development. The use of only MCQs minimizes the depth of knowledge assessment in some areas of ethics. Complete assessment of certain learning outcomes and competencies require additional validated knowledge testing methods, such as short answer questions and structured oral examinations. Additionally, we acknowledge that the relatively high number of learning outcomes in our NPM Ethics Teaching Program makes it very difficult to assess each one with a single 44-item MCQs knowledge test. By using our ranking methodology, though we ensured that the test focused on the most important learning outcomes, the test did not assess several other low ranked outcomes. Finally, we did not collect detailed demographic information regarding the pilot participants (such as their background education in ethics and clinical exposure) that could act as a confounder through its influence on the participants’ responses and opinions during the test development.

This work is part of a multi-phase research program designed to formally evaluate our teaching program. Work in progress includes the provision of the knowledge test as a pre- and post-assessment of NPM trainees’ knowledge prior to and after their participation in our NPM Ethics Teaching Program. Only three to five trainees are admitted at the University of Ottawa NPM residency program per year. Given the small sample size, data will be accumulated for at least three cohorts prior to analysis. By determining the amount of knowledge that trainees gain from our NPM Ethics Teaching Program, as educators, we will understand which concepts in the program are taught well and which ones need revision, additional teaching time, or alternative teaching methods.

Our next step includes assessing how trainees use their knowledge when navigating complex and challenging clinical situations; this is a critical learning outcome to measure. In addition to our knowledge test, we are in the process of developing a communication assessment tool for ethically sensitive scenarios that will be used during our teaching sessions but also at bedside when trainees are directly interacting with parents. We will be able to evaluate the relationship between their knowledge in neonatal ethics and their performance during these very sensitive situations.

## Conclusions

Our systematically developed knowledge test in neonatal ethics is the first published dedicated assessment tool of NPM trainees’ knowledge related to specific learning outcomes and their expected competencies in dealing with ethically challenging clinical situations. Although the test was designed around a unique teaching program, it may be useful to other medical training programs based on common learning outcomes. Future reiteration of the test will allow for evaluation and improvement of our NPM Ethics Teaching Program.

## Appendices

Neonatal-perinatal medicine ethics knowledge test

Please answer the following questions regarding your knowledge about selected topics in medical ethics.

1. The statistical approach to generating care plans in Neonatology is best described by which of the following statements:

a.     “We treat every baby because any baby may survive”

b.    “We treat all babies because it improves our standard of care”

c.     “We give parents the information and they decide”

d.    “Discussion with parents about the best interests of each baby guides us”

e.     “Based on the outcomes, we won’t treat certain babies”

2. At the end of a meeting with a pregnant mother who is at risk of delivering at the limit of viability, she asks if she could speak with her husband before making a final decision in the care for her baby. Who else could influence the mother’s decision-making process?

a.    The pediatric resident

b.    Her obstetrician

c.     Her spiritual counselor

d.    Her best friend

e.     All of the above

3. Yesterday, the senior pediatric resident had a clear, comprehensive and interactive discussion with a set of parents about their baby’s clinical condition. Today the nurse says that the parents told her that they were very dissatisfied with the meeting. What is the most likely reason for the parents’ disappointment?

a.    The senior resident did not discuss the main clinical issue

b.    The senior resident did not seek the parents’ understanding

c.     The senior resident did not recognize the parents’ emotional distress

d.    The senior resident provided direction for additional treatment

e.     The senior resident used layman’s terms when he or she talked with the parents

4. A term infant with Down syndrome (Trisomy 21) will require at least two major cardiac surgeries during early infancy for her to have a chance to survive beyond childhood. The parents have considerable ambivalence as to what to do: continue to pursue treatments that are potentially beneficial though burdensome and costly, or forego such treatments in favor of a more conservative approach. Which reason best explains why ethics is relevant in this scenario?

a.   Because the child has Down syndrome, so surgical treatment is not standard of practice

b.   Because the parents should be informed and participate in deciding what is in the child’s best interests

c.   Because there are previous cases that determine what should definitely be done in this case

d.   Because the medical team requires an ethics consult to determine the correct plan

e.   Because different opinions exist and ethics will ensure only the correct one is chosen

5. After a discussion with the parents of a severely asphyxiated baby with a grim prognosis who is unable to sustain feed orally at four weeks of age, the parents ask if NG feeds should be continued or not. Of the following options, the best one to deal with this question is:

a.  To prioritize short-term consequences over long-term consequences

b.  To suppress any underlying personal values or principles

c.  To contemplate this issue in 2-3 weeks from now

d.  To consider the benefits and limitations of each option

e.  To ask the parents if they understand the suffering of their baby

6. Which statement best describes the ethical framework(s) that guide the clinical challenge around the care of a newborn?

a.  Most decisions around the care of the newborn should be made by the mother

b.  Some of the same frameworks used for adults and older children can help guide the decisions around the care of the newborn

c.  Any decision on withholding or withdrawal of intensive care should be made only by the parents

d.  Every single clinical dilemma involving ethics can be solved using the principles of beneficence, non-maleficence, autonomy, and justice

e.  Futility is a vague principle that is best defined on a case by case basis solely by the primary physician

7. Which of the following definitions best describes ‘morality’?

a.  Culturally-dependent norms about right and wrong human conduct

b.  A body of rules of action or conduct prescribed by a controlling authority

c.  Principles that allow us to make decisions about what is right and wrong

d.  Similar cases ought to be treated in similar ways

e.  Government regulations on human conduct

8. Which model is best described by the following statement: “Rules of societal behaviour should be documented, then determine what the usual acceptable rules should be, and set up principles that will apply in a given situation.”

a.  Communicative ethics model

b.  Normative ethics model

c.  Legislative practice model

d.  Standard of care model

e.  Evidence-based medicine model

9. Which of following techniques will usually facilitate communication between the parents and the physician?

a.  Listening and judging simultaneously

b.  Considering the parents’ concerns

c.  Prioritizing your professional opinion

d.  Standing at the bedside

e.  Having five support people in the room

10. Which of the following information is most desired by mothers during an antenatal consultation at the limit of viability:

a.  Information about all complications of prematurity

b.  Information about developmental resources

c.  Information about spending time with their baby

d.  Information about different formula choices

e.  Information about neonatologists’ call schedules

11. While meeting with a mother who is pregnant at 24 weeks + 1 day gestation, you share information about only the resuscitation options and ensure that she understands what has been discussed. She is an intelligent, thoughtful woman and you are confident that she is able to choose either full or no resuscitation because she has not been inappropriately influenced by others. Though several components of the informed consent decision-making process are not yet complete, which one of the following components must be completed first?

a.  Disclosure of all relevant information

b.  Understanding of any delivered information

c.  Freedom from coercion

d.  Capacity to make a decision

e.  Informing her that she made the right decision

12. Of the following options which one is most likely the main reason parents ask the question “Have you done everything you can for my baby?”:

a.  To ensure they are not abandoned at the present time

b.  To make sure potentially dangerous treatments are tried

c.  To confirm that their physician is a kind person

d.  To demonstrate that they don’t understand the situation

e.  To indirectly force you to try at least one more thing

13. You are called to meet with the parents of a fetus who is at the limit of viability; birth will likely happen in the next few hours. After introducing yourself and ensuring that it is an appropriate moment to speak with the parents, what would usually be the best next step in your consultation?

a.  To share information about the survival rates of preterm birth

b.  To ask for consent for the care plan

c.  To ask the parents what they know about prematurity

d.  To clarify information that may influence management

e.  To share information about the risks related to preterm delivery

14. You have been asked to speak with the parents of a baby diagnosed with a complex, lethal genetic defect. The baby remains ventilated because of apneic spells. The medical team believes pursuing lifesaving treatment is futile as it causes pain, discomfort, and possible complications without any hope for improvement. After meeting with the bioethicist, the parents still wish for their baby to have full intensive care in order to maintain life at any cost. It is believed that the parents do not act in the best interest of the baby. The best next step would be to contact:

a.  The family doctor

b.  The baby’s grandparents

c.  The Court of Ontario

d.  The local provincial deputy

e.  The Children’s Aid Society

15. Which of the following ethical principles forms the basis for Informed Consent?

a.  Utility

b.  Justice

d.  Autonomy

e.  Normative

f.  Beneficence

16. “Parents have no choice but to agree to resuscitation if their baby is born at or over 26 weeks gestation”. From the viewpoint of the parents’ right to be the surrogate decision makers, this statement mostly challenges which one of the following ethical principles:

a.  Non-maleficence

b.  Beneficence

c.  Autonomy

d.  Normative

e.  Justice

17. A mother is very sad as you tell her about her baby’s lethal genetic condition. What initial strategy below will best help you address the mother’s emotion empathetically?

a.  By keeping eye contact, comforting the parent, and redirecting the conversation

b.  By acknowledging her emotion, being open, and querying what she is presently thinking

c.  By sharing personal experiences, identifying with the parent, and addressing the emotion

d.  By focusing on positive aspects, keeping eye contact, and avoiding addressing the emotion

e.  By consulting with a social worker, arranging a team meeting, and addressing the emotion

18. When meeting with a mother who is at risk to deliver at 23 weeks + 1 day gestation, she asks for information in order to make a care plan. From the physician’s perspective, which of the following is the most important piece of information to disclose:

a.  Risks of survival, cognitive delay and cerebral palsy

b.  Complications of catheter-related infections

c.  Improved survival rates with surfactant

d.  When care is considered futile after initiating NICU care

e.  Effect of socio-economic status on neurodevelopment

19. Different approaches to care for a baby in the NICU can result in differing degrees of participation by the ‘stakeholders’. The doctor is the ‘stakeholder’ with the greatest degree of responsibility for actually making the final care plan decision in which one of the following approaches:

a.  ‘Wait and See’: doctor decides when care seems futile

b.  Statistical: doctor notifies parents of plan that is based on statistical outcomes

c.  Detailed: doctor provides parents with all necessary information

d.  Individualized: doctor and parents alter plan-based infant’s best interests

e.  Integrated: doctor and parents consider infant’s best interests and society’s input

20. In your busy NICU, you are caring for two newborns. The first patient is a term newborn with a lethal condition requiring full intensive ventilatory and medical support. The second patient is a term neonate with pulmonary hypertension secondary to Meconium Aspiration Syndrome; she is also on full ventilatory and medical support. You only have enough resources to continue full support on one of these patients. Of the following ethical principles, which one best deals with prioritizing the limited resources based on the degree of benefit to the respective patients?

a.  Autonomy

b.  Non-maleficence

c.  Justice

d.  Utility

e.  Normative

21. Which of the following steps will most likely facilitate communication during the antenatal consultation?

a.  Sharing all of the statistics with the parents so they can make a proper decision

b.  Introducing yourself and sitting down before starting the interview

c.  Using touch to demonstrate empathy

d.  Bringing the mother’s chart with you to gather the details about the pregnancy

e.  Having a clear plan for the care of the infant before you leave the parents

22. A parent is considering converting the care plan for her baby from intensive care to palliative care. She asks you: “If my baby was yours, what would you do?” Which of the following is the best way to address this question:

a.  Tell them you can’t ethically answer that question

b.  Assume they’ve reached a decision and want confirmation

c.  Ask them to consider why you can’t answer that question

d.  Inquire about their understanding of the current situation

e.  Inform them about what options other parents have chosen

23. A pregnant mother was admitted to labor and delivery with preterm labor at 25 weeks + 4 days gestation. After you described the risks of being born very prematurely, the mother explains that she will not presently consent to any resuscitation for her baby; you, as the physician, are not sure if you agree. Which of the following strategies is most likely to exacerbate the conflict?

a.  Organize a multidisciplinary meeting including the obstetrician and another neonatologist to evaluate the situation

b.  Suggest that according to the Canadian Pediatric Society guideline, she has no other choice than to agree to resuscitation

c.  Try to negotiate an intervention plan that is acceptable to both parents and the medical team

d.  Explain that part of the decision-making process requires consideration of your professional duty

e.  Describe how any decision needs to be focused on the best interests of the baby

24. A physician can do one or more things to best promote early attachment and parent-child bonding in cases of a newborn with an unexpected malformation (e.g., Trisomy 21). Which of the following is the best thing to do:

a.  Examine the baby before meeting the parents

b.  Tell the parents how lucky they are to have a baby

c.  Refer to the baby by name

d.  Address only the parents’ relevant questions

e.  Remain somber at all times

25. In order to facilitate appropriate communication and understand a complex family and clinical situation (e.g., very preterm infant with parents who are Jehovah’s Witnesses), as a fellow, which of the following is the best action to take?

a.  Speak only with nursing to understand the patient’s situation and perspective

b.  Share the advice of the Division Chief with the parents regardless of its content

c.  Review the situation by considering all the social, medical, and legal issues

d.  Seek out one expert opinion that aligns with yours

e.  Decide quickly based on your own opinion what you are going to do for their baby

26. A two-week-old very preterm baby with large grade 4 intraventricular hemorrhages is dying due to severe necrotizing enterocolitis. The medical team believes palliative care is now in the best interests of the baby but a possibly life-saving laparotomy could be performed. Which of the following scenarios is most likely to lead to potential conflict when determining the care plan with the parents?

a.  The parents are not affiliated to any specific religious beliefs

b.  The parents agree with the medical team’s prognosis

c.  The parents are willing to contemplate a decision in six weeks

d.  The parents want to ensure there is no suffering

e.  The parents have a high level of understanding

27. After parents are fully informed and have chosen a care plan for a possible premature delivery, what is typically the next best step to take?

a.  To share all of the information regarding survival, morbidity, and long-term outcome

b.  To give the parents time to reflect together

c.  To ensure that they understand the information that you have shared

d.  To review in detail what will happen to the baby after birth

e.  To confirm their decision and support it

28. The parents of a baby boy bring to your attention the fact that they will not consent to the end-of-life decision for their severely compromised newborn baby. You decide to proceed with extubation and palliative care. This situation best demonstrates disregard to which one of the following ethical principles?

a.  Justice

b.  Non-maleficence

c.  Medical futility

d.  Baby’s best interests

e.  Parental authority

29. In regards to the decision-making process at the limit of viability, which of the following statements is the most appropriate?

a.  Most parents want to decide by themselves

b.  The vast majority of parents want to make the final decision

c.  The majority of parents want the doctor to make the final decision

d.  The majority of parents want to participate, but at differing levels

e.  Most parents want the bioethics committee to be involved

30. What is the most reasonable first step when approaching an ethical problem?

a.  Notify the Children’s Aid Society

b.  Determine the relevant issues

c.  Consult the ethics team

d.  Leave the decision-making to the parents

e.  Discuss the case with colleagues

31. Parents need to make decisions that are in the best interests of their child. Which of the following is most likely to limit their objectivity?

a.  A lack of sleep resulting in fatigue

b.  A desire for a perfect child

c.  An understanding of community supports

d.  A large number of children

e.  A stable job with good income

32. A premature baby needs a blood transfusion because she is anemic, septic, and may die in the coming hours without the transfusion. While speaking with the nurse, she informs you that the parents are Jehovah’s Witnesses. Which one of the following relevant options will take precedence in determining whether or not to proceed with transfusion?

a.  Society’s values

b.  Patient’s best interests

c.  Parents’ religion

d.  Physician’s beliefs

e.  Parents’ consent

33. In a case where the parents don’t want to be with their baby before he or she dies, as a physician, what would be the most appropriate initial response for you to say to the parents?

a.  “It is better for you to be with your baby – it will help you with the grief process.”

b.  “Are you able to tell me why you don’t want to be with your baby?”

c.  “We are family-centered here, so we encourage you to stay with your baby.”

d.  “Okay, that is okay, you don’t need to be with your baby.”

e.  “Sorry, but you cannot let your baby die alone in the NICU.”

34. After setting up the interview, what is the best next step for communicating bad news to parents?

a.  Determining the parents’ perception of the situation

b.  Describing the important medical issues

c.  Summarizing the results of all investigations

d.  Acknowledging your own perception of the situation

e.  Strategizing as to the next steps in management

35. A mother presents to the local community hospital at 24 weeks + 1 day gestational age. She has ruptured her membranes and is in labour. The obstetrician tells them that due to the extreme prematurity and the fact that she is almost certainly going to deliver soon, the chance of having a surviving baby with no or only mild impairments is less than 25%. Therefore, the obstetrician says no interventions for the mom or baby will occur as this is in the best interests of the infant. Which ethics model of physician-patient relationship does the above scenario best demonstrate?

a.  Informative: physician gives information and intervenes as per the parents’ decision

b.  Deliberate: physician gives information and helps parents choose the most important values

c.  Paternalistic: physician gives information and provides care based on what she/he thinks is best

d.  Shared: physician fully incorporates parents in the decision-making process and after deliberation, respects their decision

e.  Interpretive: physician gives information and elucidates parents’ values about the situation so they can make a decision

36. When confronted with a conflict between the medical team and the parents about continuing intensive care versus transitioning to palliative care, what is the most important question to ask yourself to ultimately resolve this conflict?

a.  Will I be in trouble as a professional if I do what the parents ask?

b.  Are the parents’ wishes medically appropriate?

c.  How will my colleagues judge my decision?

d.  Could I be sued by the parents?

e.  Do the parents’ desires conflict with my personal values?

37. Which of the following elements is part of the informed consent decision-making process?

a.  Assuming parents are competent

b.  Disclosing all information

c.  Hoping the parents understand what you communicated to them

d.  Asking the parents to immediately agree on a proposed resuscitation plan

e.  Ensuring you attempt to influence the parents’ decision

38.  A 41-year-old mother, pregnant after in vitro fertilization, is admitted with ruptured membranes. She is pregnant at 21 weeks + 4 days gestation. Of the following pieces of history, which one is most likely to result in the parents arguing for full resuscitation?

a.  The mother is a school teacher

b.  The mother has been having bleeding on and off for four weeks

c.  The mother is on an antidepressant

d.  The mother is gravida five with four previous miscarriages

e.  The mother has three siblings who live out of town

39. An infant is born with a hypoplastic left heart. A cardiac surgeon provides all the information about the malformation and its consequences. The parents understand it all. The surgeon tells them that the decision to either do surgery (or transplant) or provide palliative care belongs to them. She tells them to think about it over the next 48 hours and then she will come back to hear their decision and make a clear treatment plan for their infant. Which classic approach to care in neonatology does the above scenario best demonstrate?

a.  ‘Wait and See’: doctor decides when care seems futile

b.  Statistical: doctor notifies parents of plan that is based on statistical outcomes

c.  Detailed: doctor provides parents with all necessary information

d.  Individualized: doctor and parents alter plan-based infant’s best interests

e.  Integrated: doctor and parents consider infant’s best interests and society’s input

40. Which one of the following factors that influence the decision-making context occurs most frequently in the setting of any neonatal case?

a.  The ability to do something means it should be done

b.  The infant cannot speak for him/herself

c.  The prognosis is certain

d.  The best interests of the infant are not yet relevant

e.  The parents are able to think clearly

41. When interacting with the consultant during an antenatal consultation at the limit of viability, which of the following characteristics would a mother most likely identify as a strength:

a.  The consultant gave me a chance to talk about my concerns

b.  The consultant answered my questions in medical words

c.  The consultant gave me a chance to ask questions once

d.  The consultant made sure that there was no uncertainty

e.  The consultant assumed that I understood the information

42. Of the following statements, which one best describes parental involvement in the decision-making process for the care of their baby who is at risk of being born at the limit of viability?

a.  Parents almost always prefer that the physician make the decision for them

b.  Parents who want to be involved may not be identified by physicians

c.  Parents rarely need physicians’ advice during the process

d.  Parents generally find the decision-making process easy

e.  Parents minimize involvement so they don’t impair attachment to their baby

43. A set of parents are making a difficult decision around whether to change to a palliative care plan for their critically ill neonate. They understand the clinical situation well. They recognize their values that are relevant to making the present decision, but they cannot reach a final decision. Which of the following ethical models of the physician-patient relationship would be best to use?

a.  Informative: physician gives information and intervenes as per the parents’ decision

b.  Deliberate: physician gives information and helps parents choose the most important values

c.  Paternalistic: physician gives information and provides care based on what she/he thinks is best

d.  Shared: physician fully incorporates parents in the decision-making process and after deliberation, respects their decision

e.  Interpretive: physician gives information and elucidates parents’ values about the situation so they can make a decision

44. The fact that a neonate cannot speak for him/herself means that someone else has to consider his/her best interests. Of the following, the value to consider first when making decisions on his/her behalf is:

a.  The potential impact on siblings

b.  The religious beliefs of the parents

c.  The importance of life itself

d.  The parents’ well thought out desires

e.  The effect of a death on parents
